# The Moral Gatekeeper: Soccer and Technology, the Case of Video Assistant Referee (VAR)

**DOI:** 10.3389/fpsyg.2020.613469

**Published:** 2021-01-12

**Authors:** Ilan Tamir, Michael Bar-eli

**Affiliations:** ^1^School of Communication, Ariel University, Ariel, Israel; ^2^Business Administration Department, BGU, Ben-Gurion University of the Negev, Beersheba, Israel

**Keywords:** sport, soccer, technology, VAR, moral

## Abstract

Video assistant referee was officially introduced into soccer regulations in 2018, after many years in which referee errors were justified as being “part of the game.” The technology’s penetration into the soccer field was accompanied by concerns and much criticism that, to a large degree, continues to be voiced with frequency. This paper argues that, despite fierce objections and extensive criticism, VAR represents an important revision in modern professional soccer, and moreover, it completes a moral revolution in the evolution of the sport as a whole. Theoretically speaking, this technology enables an improvement in the sport’s professional standards and its public image and prestige, and especially its moral standards – Fair play. Furthermore, the introduction of this technology makes it possible to discover additional weaknesses (Standardization for extra time, a clear definition of a handball offense and more) that professional soccer regulations will probably be forced to address in the future.

## Referees’ Mistakes in Sport

A central ideal of sport competitions is expressed by the traditional notion of “let the best athlete or team win.” That is to say sports governors should try minimize as much as possible mistakes and falsification from deciding who the winner is. In order to increase the probability that this will indeed be the case, referees are installed in order to ensure fair competition. In soccer (and other sports as well; see MacMahon et al., 2015) their possible influence on the outcome of games (and the safety of players) is immense. For example, at least two World-Cup finals were decided by controversial decisions of the referees.

On July 30, 1966, England and West-Germany competed at the Wembley stadium in London in the final game of the World cup. With 11 min of extra time gone and a tied score of 2:2, English striker Geoff Hurst received a cross, swiveled and shot from close range. The ball hit the underside of the crossbar and bounced down. Referee Gottfried Dienst was uncertain, but awarded a goal to England after consulting linesman Tofiq Bahramov from Azerbaijan in the USSR, who indicated that it was a goal. The game ended 4:2 for England and this decision has remained controversial ever since then. Furthermore, it led to the creation of the expression “Wembley goal,” a phrase used to describe any “Ghost-“or “Phantom-goal” awarded (but actually not scored) in a similar fashion.

On July 7, 1974, West-Germany and Holland competed at the Olympic stadium in Munich in the final game of the World Cup. In the 25th min, when the score was 1:0 in favor of Holland, German striker Bernd Hoelzenbein received the ball at about 40 m diagonally from the Dutch goal, and started to dribble. While entering the penalty area, he was tackled by the Dutch defender Wim Jansen, and fell down. The English referee, Jack Taylor, awarded a penalty kick to West Germany, which was used by Paul Breitner to tie the game, and the Germans won 2:1. This crucial referee’s decision remains controversial until today due to the fact that Hoelzenbein was accused of being dishonest and faking the collision, which he vehemently denied (Sabag et al., 2018). This example involves player deception and is a judgment call by the referee after witnessing an event.

Although these are two different examples, is an eye-witness error and the other a judgment decision which involves a more complicated situation, in both cases the truth remained in doubt.

Bar-Eli (1994) was appointed psychological consultant to the Israel soccer referees association. One major purpose of this intervention was to help referees improve their performances. While discussing the issue with Abraham Klein, an experienced, highly respected international ex-referee (considered by many as the best Israeli referee ever), who served at that time as chairperson of the association, these two cases were discussed. Bar-Eli argued that soccer would only profit from [A] introducing a goal-line camera to decide whether “the whole of the ball passes over the goal line” (as the laws of the game require for approving a goal) or only 97% of the ball (as more recent studies indicate with regard to Hurst’s “Wembley Goal”; see, for example, Reid and Zisserman, 1996); [B] using some other available technology, to make better decisions in ambiguous tackling situations (e.g., such as where a penalty kick should eventually be awarded).

Bar-Eli contended that the use of such technologies (i.e., decision aids) will INCREASE the referee’s chances to ensure the realization of the very basic notion of “may the best athlete or team win,” thereby STREGTHENING the referee’s authority on field.

Referee Klein strongly objected to Bar-Eli’s suggestions, arguing that the referee’s authority on the field will be damaged (at least at the perceived level) as a result of applying such technologies – even with regard to the simple goal-line decision, not to mention the more complex penalty kick situation. However, ongoing controversies such as the abovementioned ones (i.e., from the World Cup finals of 1966 and 1974), and/or other extreme, decisive referees’ errors such as in the case of Maradona’s notorious ‘Hand of God’ affair in the 1986 World Cup, accelerated research on referees in soccer (and umpires, officials, linesmen or judges in other sports). Raab and Helsen (2015) argued that referees’ performances are determined by physical (e.g., the position of the referee) and perceptual-cognitive (e.g., interpretation of events on the field) factors. They identified an increasing number of publications focusing on physical (*n* = 67) and perceptual-cognitive (*n* = 58) aspects of refereeing between 2000 and 2013. Pina et al. (2018) more recent review revealed a similar trend for publications focusing on physical (*n* = 74) and perceptual-cognitive (*n* = 90) aspects of refereeing between 2000 and 2016, but with a greater emphasis on perceptual-cognitive aspects such as judgment and decision making (JDM) and perspectival bias (see also Helsen et al., 2019).

The growing interest in researching referees’ JDM reflect a “psychology OF sport”- perspective that is, the use of psychological knowledge to cope with a practical problem, in this case, referees’ mistakes. This view acknowledges the commercial, media and financial interests in the business of soccer (and sport in general), in which a sound referees’ JDM is required to minimize the negative effects of erroneous officiating decisions on match outcomes (Helsen et al., 2019). However, the increasing interests in referees reflect also a “psychology IN sport”-perspective, that is, a view which contends that “studying sport is a great idea, because people make many decisions that matter enormously to them under standard conditions. It is actually one of the places to do this” (Kahneman, 2008). Indeed, referees’ JDM has been increasingly viewed as one of the best fields to study human JDM processes in general, as evident from Nobel-Prize winner Daniel Kahneman’s quote, and also from earlier reviews of JDM in sport (Bar-Eli et al., 2011) as well as from more recent ones (Raab et al., 2019a,b).

Sport fields are not environments that are conducive to every JDM process. This is true for all the parties involved, from players to coaches, and especially referees. In soccer, the rapid movements of the ball and the players, the varying sight lines, and the heated passions, pressure, and competition expose referees to very complex, sometimes impossible, officiating situations. More specifically, top-referees are conceived as experts who make decisions in dynamic, time-constrained sporting environments (Raab and Helsen, 2015). Many studies from countries around the world that have examined JDM of referees in a wide range of sports, as will be detailed later, have found that referee JDM are affected by significant biases that directly affect games and their results.

## Psychological Biases

As mentioned above, much of the “raison d’etre” of the “psychology OF sport”-approach toward the study of referees’ JDM, reflects an explicit or implicit intent of trying to minimize the probability of referees’ mistakes. Accordingly, this literature has been quite focused on the concept of bias, which was defined as distortion of measurement or evaluation results leading to their misinterpretation (Helsen et al., 2019; Raab et al., 2019a).

However, the very notion of “bias” requires a benchmark of “something which is NOT biased,” to be compared with. In other words, we have to clarify what a GOOD decision of a referee actually is.

At first glance, this seems quite easy, because the primary, very essential reason of using referees at all, would be the enforcement and interpretation of the laws of the game, which motivates the referees to be as ACCURATE as possible. This would mean that if the referee is inaccurate, he or she may be biased in some way or another. However, Bar-Eli et al. (2011) – based on previous work conducted by Plessner and his team – maintain that soccer referees can also conduct GAME MANAGENENT, which is intended to ensure the flow of the game and be (or at least appear) unbiased, with accuracy considered less important than “just return home safely” (i.e., officiating a game without any noticeable incidents). These two strategies may point to the same direction, but they can also get into conflict. Thus, while most of the research on referees’ biases is concerned with (in)accurate JDM, referees should also be investigated as to the ways in which they adjust their interpretation of incidents to the concrete context of the situation in question (Bar-Eli et al., 2011). The basic premise, therefore, is that increasing the use of objective, seemingly accurate (technological) means will help reduce biases and increase fairness.

More recently, Bar-Eli (2018) reviewed some of his own work on referees (though mainly in basketball) and concluded that in general, referees call fewer fouls than those judged by (basketball) experts as deserving a call. This “conservative” behavior can be explained both by rational reasons and biased JDM. The best objective referees’ desire is that nobody question the accuracy and fairness of their calls in the contest. This strategy makes officiating mistakes and interpretations, not only understandable, but also, though paradoxically, rational.

Similar trends were revealed among soccer referees (e.g., Sabag et al., 2018), who strive for accuracy and are also aware of the severe consequences of player dismissals (i.e., red cards; see Bar-Eli et al., 2006) – Referees sometimes try to avoid using measures such as yellow or red cards too soon in the game, thereby producing a possible escalation in which too many dismissals would ruin the game (and the respective TV-ratings and broadcasting income). These considerations leave us again with the open question of what can be considered good referee decisions (Bar-Eli et al., 2011).

Many variables naturally affect decision making in any area. The field of sports, however, accommodates an extraordinary concentration of passions, pressures, and emotions (Hanin, 2007), whose effect on referee JDM process is direct and immediate. One of the manifestations of this effect is compensating bias, which is when referees who make decisions in favor of one team try to even out the situation in subsequent decisions. These referees will impose more stringent criteria against the team that won the previous call (on an offense, sending off a player, etc.) and will lower the criteria when calling against a rival.

A study conducted in Germany (Schwarz, 2011), which examined the penalties in the local soccer league over four decades, is an example of numerous studies that found proof of compensating bias. In the majority of games with two or more penalty kicks, the calls were divided equally in favor of both teams. The referees effectively raised their criteria for awarding a second penalty to the team that already received a penalty call, and relaxed the criteria for the opposing team. The study also examined the timing of the whistle for penalty kicks and found that when two consecutive penalty kicks were awarded to two different teams, the interval between the referees’ calls was significantly shorter than when two consecutive penalty kicks were awarded to the same team.

Studies on the NCAA, the US college basketball league (Anderson and Pierce, 2009; Noecker and Roback, 2012) found the exact same pattern: Referees tend to call more fouls against the team that has fewer penalties in order to even out the competition. Moreover, referees also call more penalties against the team that is leading. A similar study (Plessner and Betsch, 2001) found that a referee’s previous call affects his subsequent decisions in the game, reflecting a tendency to even out his decisions in the game. The expectation from the referees in the game therefore is to whistle according to the events, without taking into account extraneous considerations, which can impair the accuracy of the decisions.

Another manifestation of the psychological effects that influence referees is related to reputation or prior knowledge bias. The reputation of a team or a player affects referees’ calls during a game. The assumption that a referee can ignore all the previous knowledge he has of the players is not borne out by evidence. A study by Jones et al. (2002) illustrates this idea: 38 soccer referees viewed several video clips of segments of taped soccer matches. The same team (wearing a blue uniform) appeared in all the clips, playing against a different rival in each case. The referees were divided into two groups and were instructed to describe the call they would make in each case. The instruction sheet handed to one group contained a comment that the blue team is considered an aggressive team. Interestingly, while no differences emerged in the decisions of the referees in both groups (e.g., the number of fouls they called), the groups differed in the interpretation that they gave to each event. The referees who had prior knowledge of the blue team’s “past” awarded more yellow and red cards than the other referees. In other words, referees’ previous knowledge of the team directly informed their decision making.

Referees are subject to many more psychological effects, including the pressure imposed on them by the crowd (e.g., Nevill et al., 2002), which has been shown to affect decisions including stoppage time (Garicano et al., 2005; Scoppa, 2008), penalty awards (Dohmen, 2008), and red and yellow carding (Buraimo et al., 2012).

Some effects are less intuitive, for several years, researchers have noted that the color of players’ uniforms affect their decisions. For example, red has a positive impact on the outcome of a contest. The fact that contestants are more successful when they wear red has been proven in a broad range of competitions and sports (Hill and Barton, 2005). According to one explanation, cultural and evolutionary variables link the color red to dominance and aggression and thereby psychologically affect contestants (Elliot and Maier, 2014; Meier et al., 2015). Namely, wearing red enhances one’s dominance, aggressiveness, and testosterone, which facilitates competitive outcomes. Ilie et al. (2008) even recognized highly significant effect on the performance of red teams in a popular multiplayer first-person-shooter (FPS) computer game. This effect of course does not apply to soccer referees or result of their decisions.

Referees are also affected by the colors that players wear. One study conducted recently (Hagemann et al., 2008) demonstrated how referees (in this case, in taekwando), give higher scores to competitors who wore the color red. One of the researchers’ conclusions was that the prominence of specific colors, and especially red, allow referees to more easily assess the many moves that players in these colors perform. In contrast to other colors that blend into the background and conceal players’ moves from the referee’s notice, the color red attracts the referee’s attention and affects his partiality. Similar findings have also been reported in the England soccer leagues (Attrill et al., 2008; Olde Rikkert et al., 2015), in the Turkish soccer league (Tiryaki, 2005) and more.

## Physical Limitations

Many sports events are inherently dynamic. Motion and speed are frequently the main elements of a game, and it has become almost impossible for a referee to attend to all the events occurring on the field (the single referee in soccer is certainly a problem toward achieving good officiating decisions). As a result, some sports have multiple referees while other sports use linesmen or a crew of assistant referees, for example. But does the added assistance always meet the challenges of decision making? Not necessarily. Sometimes the angle of a referee’s sight of the ball, the players, or other assistant referees, can lead to error. Studies that examined the location of linesmen on the soccer field found that errors regarding offside calls were almost inevitable (Mallo et al., 2012). Studies from various countries indicate that between 17 and 25% of all decisions made by referees and linesmen in a game are inaccurate (Gulec et al., 2018).

The implications are enormous. Linesmen make dozens of calls in every game, and for every ten offside decisions, one or two calls are in error. The error of failing to raise a flag (when the linesman should signal for an offside) occurs more frequently than the error of raising the wrong flag (Mallo et al., 2012). The VAR in this case can be of great help when it oversees the decisions of the linesmen (approve or disqualify a goal as a result of an offside for example; approve or disqualify a penalty kick called by the linesmen, etc.). When we look at the errors made by referees, the number is even larger, although referees are less restricted in their position on the field than linesmen, and a share of these errors can be prevented by improving referees’ physical condition and positioning. In any case, referee errors have become such an integral part of the game that the term “ghost goal” has become common in soccer parlance, and is used to express the many cases of debatable or questionable goals (Similar to other examples like “Hand of God” and others).

## Sports and Technology

Although the integration of technology in sports, and specifically technology designed to support referee decision making, is warranted in view of the distortions described above, it seems that sports has assumed a double role with respect to technology (Tamir, 2019). On the one hand, the sports industry is leading significant technological revolutions. This is certainly true in the field of media (Galily and Tamir, 2014). Sports is considered a major agent in the introduction of various technologies including plasma screens and HD- and 4K-quality broadcasts in the home. On the other hand, sports’ religious-like devotion and commitment to its communities and traditions (Bain-Selbo and Sapp, 2016) highlight the conservative elements of the industry and inhibit new technology adoption.

Loland (2002) argues that the integration of technology into sports should be assessed through one of the following three perspectives, based on the defined aim of sports: If sports is a means to achieve external goals such as political, ideological, or financial prestige, technology’s significance lies in its ability to achieve these goals (by creating manipulation and giving an advantage to the athlete). Moral issues related to technological integration do not appear to be relevant in such a case. The history and role of sports in the Cold War (Dimeo, 2007) or, alternatively, in contemporary economic rivalries (Simon et al., 2015) illustrate the relevance of this argument.

If sports is a platform for realizing physical potential, technology’s significance lies in its ability to help individuals improve their performance. In this case, too, history indicates that many athletes and sports professionals have used this argument to support the use of prohibited substances. At the same time, within the context of this goal, technology should ensure standardization of performance and ensure balance, credibility, and validity of assessments, evaluations, and judgments.

The third perspective takes a broader approach and views sports as an arena of potential human development. Sports is a sphere with a set of values and encompasses more general human virtues and merits, and the moral virtues of human development. Sports focuses on normative values and behaviors (as respect for the rules of the game and the competitors), and Pierre de Courbrtin’s view on reviving the Olympic Games is perhaps emblematic of this view (MacAloon, 2013). In contrast to the previous approaches, which tend to accept any technology that is instrumental in achieving an external goal or enhancing performance, this perspective assumes a clearly moral stance that emphasizes the journey. Technology might lead to better outcomes but if the athlete is unable to control it, it does not have a value. Improving performance without athletic effort has no value.

The involvement of technology in sports can be generally divided into two: technologies that help promotes athletes’ achievements and technologies that are used as a governing mechanism of sport. The performance development range is very wide, from body suits in swimming designed to reduced water friction, or shorter alpine skis with radically improved carving capabilities to potentially performance-enhancing genetic technologies. Fundamental resistance to the introduction of new technologies into sports, in this context, is related to the physical component in the definition of sport. That is to say, according to critics, it is the human body and not a machine that should be trained to overcome challenges and natural attributes (Fouché, 2017).

In the second category, many innovations that have penetrated the various sports industries in recent years can be identified, as will be detailed below, mainly with the aim of assisting the referees and regulatory process.

Of course there is a connection between the two, and many times the better the athletic performance the harder it is to make good calls, because of the increased speed and skill (in many sports and certainly in soccer).

## Technology and Refereeing

Technology’s penetration into sports, as a governing mechanism, has been clearly felt in recent decades (Fouché, 2017), creating a significant impact on the entire spectator experience (Dyer, 2015). If the basic assumption was always that the match officials were close to the events and had a better view than anyone else (for example, the high position on the tennis court), the age of television changed the rules (Collins, 2019). The high quality broadcasts and the television replays may put the TV viewers in a better position than the referee when it comes to identifying actions and situations. Naturally, this reality has created a growing sense of unease and distrust among viewers. Therefore, it was only a matter of time before the gaps narrowed. And so, replays (Collins, 2010), photo finishes, goal-line technology, hawk-eye systems, and other technologies, designed to assist the referee, have transformed sports into a more accurate space than before. Some of the technologies provide autonomous assistance, which means giving an indication to the referee during the game (such as in rugby, or in some cases in cricket), and some based on player challenge (such as tennis). As technology will improve, the second type is likely to approach the first, and in each case the intention is, in both cases, to use the technology to reduce the errors of referees and return viewers’ confidence in the sport. Although, the introduction of new technology was accompanied by criticism (Dyer, 2015).

For example, the aspiration and expectation of perfect accuracy from the technologically assisted is probably impossible (Collins, 2019). In part, because the lines drawn on sports fields and the edges of balls are not perfectly defined (p. 21) but more importantly, because the measuring devices are based on the world of virtual reality, not the actuality of a physical world. The technology do not show what happened, but a statistical assessment of what probably happened (devices such as ball-trackers, not showing what actually happened but only a statistical estimate of what might have happened) (p. 25).

Another criticism is related to the continuity of the game. The argument is that the use of technology takes a long time to decide and impedes the flow of the game. The frequent stops are tedious and make the game, exhausting and damage the entertainment component of the game (Ryall, 2012). Collins (2019) argues that the guiding principle is to play the game with technology as close as possible to the game without the technology.

Alternatively, some also claim that the use of technology is also not always being correctly applied, because there are some sport situations whereby technology cannot conclusively affirm a correct decision. Some decisions, such as whether the ball crossed the goal line or not, are allegedly simply right or wrong. Immediate and accurate goal technology seems to be a clear step forward. Other decisions involving referee judgment, for instance on player intention and potential sabotage of the game (such as decisions on ‘professional fouls’ and yellow or red cards), may be more complicated. One might argue that they are best made in the flow and full context of the game, and that video replays, sometimes in slow motion, can lead to misinterpretations.

## Soccer and VAR

Video assistant referee technology is an example of autonomous assistance given to soccer referees. Although VAR was tested for the first time during the 2012–2013 season, it was officially introduced into the Laws of the Game in 2018 to help referees in reviewing decisions made by the head referee by means of video footage only for three main situations and one administrative incident (Lago-Peñas et al., 2019).

According to the IFAB (International Football Association Board), Principles, a video assistant referee (VAR) is a match official, with independent access to match footage, who may assist the referee only in the event of a ‘clear and obvious error’ or ‘serious missed incident’ in relation to:

(a) Goal/no goal; (b) Penalty/no penalty; (c) Direct red card (not second yellow card/caution); and (d) Mistaken identity (when the referee cautions or sends off the wrong player of the offending team). The original decision given by the referee will not be changed unless the video review clearly shows that the decision was a ‘clear and obvious error.’ The final decision is always taken by the referee, either based on information from the VAR or after the referee has undertaken an ‘on field review’ (OFR).

In soccer, the introduction of the new technology has been the target of intense criticism. Top soccer executives including former FIFA President Sepp Blatter, voiced their objections to the integration of advanced technologies into soccer games (CBC Sports, 2008), echoing objections made against previous technologies designed to improve the game, such as goal-line technology (Ryall, 2012). The source of opposition in all cases was soccer executives’ stance on the nature and authenticity of the game. Their basic assumption was that soccer is the most popular sports in the world due to its simplicity and authenticity. The introduction of technology, they argued, would undermine the deep roots of the most popular game in the world (Walsh, 2011). In other words, human errors are an inevitable part of the game, and even part of its charm. Other objections warned against the time that would be wasted in the game as a result of repeated viewing of video-recorded moves; like other real-time video-replay devices, the criticism of VAR was the possible disruption to the flow and pace of the game due to the stopping and starting (Dyer, 2015). These interruptions to the flow of the game were expected to cause viewers to lose interest. However, according to the FIFA website (FIFA, 2018), technology actually reduces wasted time (time that was apparently taken in the past for arguments with referees). Other critics were concerned about the potential damage to referees’ authority (Collins, 2010), and pointed to the large number of penalties in the most recent 2018 World Cup Games in Russia to illustrate how technology changes the game for the worse (29 penalties, more than twice the number of the penalties in the Brazil 2014 games).

Eventually, after years of dispute, soccer executives realized that it was no longer possible to deny that the era of multi-camera HD capture systems and broadcasts had arrived. As viewers at home see each move and referee error in replays showing various angles, soccer and professional leagues should welcome VAR to restore supreme value of the game, namely, fair play. It is important to emphasize that officiating technology will not eliminate all referee errors in sport. As long as human beings are referees there will be officiating mistakes. However, the VAR will help referees make better decisions and rule interpretations, will lessen unfairness, and can be endorsed by the soccer community.

## Conclusion

The new referee system offers a dramatic contribution to the game of soccer. In Loland’s (2002) terms, it gives referees the prestige and honor eroded over the years. The VAR system clearly improves professional decision making during a game, and reinstates in soccer the supreme value of fairness and fair play. After years in which the average viewer saw a large number of referee errors due the fallibility of referees, the VAR has managed to improve fair conditions sought by referees and viewers. From a professional perspective, the VAR system promotes limited impartial and accurate decision making. Although the system does not clarify all potential areas of ambiguity, reviewing a video replay reduces errors and accordingly enhances the professional standards of both referees and players.

Incidents in which players significantly “dive” to obtain a penalty kick or other unprofessional offenses are committed will gradually be reduced (applies to accurate eye-witness reports, and not referee judgments and game management aspects of officiating) and filter out the game’s distracting background noises. However, perhaps the most important point is the impact of technology on the moral aspects of the game in relation to human behavior. Fair play is a revered principle of sports. Without ensuring equal chances and fair judgments, sport is sawing off the branch on which it is sitting. That is, it must invest in and make use of technological resources to monitor and enforce the principle of fairness. When circumstances limit the capabilities of referees, especially due to physical limitations, errors could be accepted and contained. However, according to the technological determinism theory (McLuhan, 1964), any new technology that penetrates an industry dictates new standards for evaluating reality, and this is the case for sports (in a limited sense—as far as what cameras focus on and is aired). From the moment viewers at home see moves clearly and sharply, they often voice their disapproval of referees’ calls and criticize soccer associations for their errors, demanding accuracy. Officiating technology has the potential to change the game’s values and make it more accurate and fairer. Still, the use of VAR is selective, perspectival, two-dimensional and not fully all-encompassing, thus technology does not rule out the efficacy of on-field referees to make accurate and fair decisions.

The argument of the present article is that the use of technologies in sports should be examined in the light of morality and as such, the VAR system, which receives (unsurprisingly) much resistance, should be treated as one of the most important and moral changes in soccer. At the same time, it is important to remember that change in moral conduct requires an internalization by human beings to comprehend wrongfulness, willfully alter their attitudes and then express right actions in sport to the best of their ability.

It is further important to recall that VAR operates under the Laws of the Game. Beyond accuracy, the system also has the potential to reveal additional weaknesses in the Law of the Game that have become integrated into the fabric of acceptable errors (e.g., hand touches, time measurement and other factors).

A good example might be stoppage time in soccer. Stoppage time is an important issue because it can account for more than 10% of the total game time. Studies show that there is not always a connection between the number of minutes a referee decides to add and the time the play was actually stopped (Lago-Peñas and Gómez-López, 2016). Today, as the subjective aspects of decision making have been reduced, and accuracy has become a top priority, stoppage time is yet another issue that can be quantified and redefined through technology.

The introduction of advanced technology in professional soccer has and will continue to significantly improve the game. This is an important step forward for soccer matches and an advancement for all sports. Collins (2019) argues that if referee decisions seem reasonable to both the human eye and television viewers on replay, then a sense of unfairness can be reduced and lessen the annoyance for example of stoppage time inconsistency (based on the principle of continuity). Despite justifiable criticism, the integration of technology in sports to promote the principle of fair play should be encouraged. In the case of soccer, the VAR will increase fairness in officiating and raise the level of impartiality with referee decisions.

## Author Contributions

Both authors listed have made a substantial, direct and intellectual contribution to the work, and approved it for publication.

## Conflict of Interest

The authors declare that the research was conducted in the absence of any commercial or financial relationships that could be construed as a potential conflict of interest.
